# Clinical implication of ambulatory milrinone therapy in patients with advanced heart failure

**DOI:** 10.1002/clc.23953

**Published:** 2022-11-20

**Authors:** Yuri Nakagawa, Teruhiko Imamura

**Affiliations:** ^1^ Second Department of Internal Medicine University of Toyama Toyama Japan

To the Editor,

1

The clinical implication of ambulatory intermittent inotropes therapy is receiving great concern thus far given that an incremental number of patients refractory to guideline‐directed medical therapy (GDMT) are not candidates for cardiac replacement therapy. Laufer‐Perl et al. demonstrated that repetitive, intermittent, and short‐term milrinone therapy was safe and potentially efficacious in ambulatory patients with advanced heart failure who were intolerant to maximum GDMT.^1^ Several concerns have been raised.

In their study, participants received IV furosemide and IV iron supplementation if necessary during routine clinic visits concomitantly to milrinone therapy.^1^ To exclude their impact on clinical outcomes, we would like to confirm whether these therapies were performed before the milrinone therapy.

In general, those with advanced heart failure complaining of New York Heart Association functional Class IIIb–IV indicate in‐hospital treatment. Were there any critical reasons why they were not hospitalized and remained as outpatients?

In their study, many patients had intolerance to maximum GDMT at baseline.^1^ Following the initiation of milrinone therapy, the anti‐heart failure agents could be uptitrated. However, patients seemed to have relatively preserved blood pressure (median systolic blood pressure was 117 mmHg) at baseline. Could the authors show the criteria of how to titrate anti‐heart failure agents? Anti‐heart failure agents might have been further uptitrated without milrinone therapy.

According to the lists of medications,^1^ further attempt to uptitrate anti‐heart failure agents could have been conducted against symptomatic hypotension and chronic kidney diseases, including ivabradine, vericiguat, and percutaneous valvular intervention if applicable, all of which do not affect blood pressure considerably. Did the authors consider these medications/interventions? Also, could the participants up‐titrate the dose of beta‐blocker following the initiation of milrinone therapy?

The prescription of SGLT2 inhibitor was significantly increased following the initiation of milrinone therapy. However, given that SGLT2 inhibitors have very recently been recognized as one of the GDMT, their incremental prescription rate might have been due to incremental awareness and willingness of the physicians rather than the stabilized hemodynamics by milrinone.

## Data Availability

NA.
